# Supplementary material for: Observation of negative capacitance in antiferroelectric PbZrO3 Films
Source: Nat Commun. 2021 Jul 9;12:4215. doi: 10.1038/s41467-021-24530-w (PMC8270919; doi:10.1038/s41467-021-24530-w)
Supplement: Supplementary file 1 — Supplementary Information [file 41467_2021_24530_MOESM1_ESM.pdf]

Supplementary Information for

Observation of Negative Capacitance in Antiferroelectric Film $PbZrO_3$

Leilei Qiao[1], Cheng Song[1*], Yiming Sun[1], Muhammad Umer Fayaz[1], Tianqi Lu[2], Siqi Yin[1], Chong Chen[1], Huiping Xu[1], Tian-Ling Ren[2], Feng Pan[1*]

[1]Key Laboratory of Advanced Materials (MOE), School of Materials Science and Engineering, Tsinghua University, Beijing 100084, China

[2]Institute of Microelectronics & Beijing National Research Center for Information Science and Technology (BNRist), Tsinghua University, Beijing 100084, China

*songcheng@mail.tsinghua.edu.cn; panf@mail.tsinghua.edu.cn

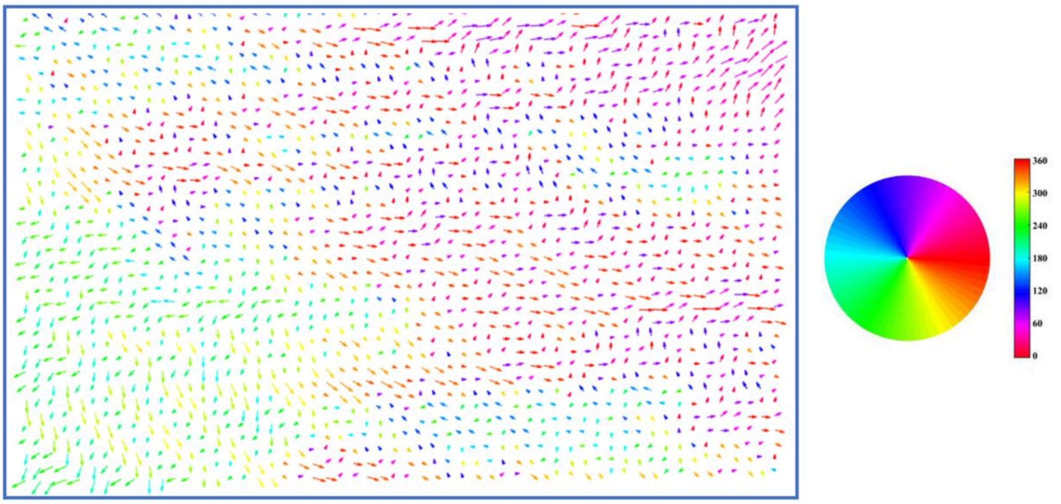

Supplementary Fig. 1 Pb-cation displacement vector maps revealing the two antiferroelectric domains with different orientations. The different colors represent the different polarization directions, as shown by the scale bar on the right. It is obvious that the left lower vectors have different orientations with those on the right.

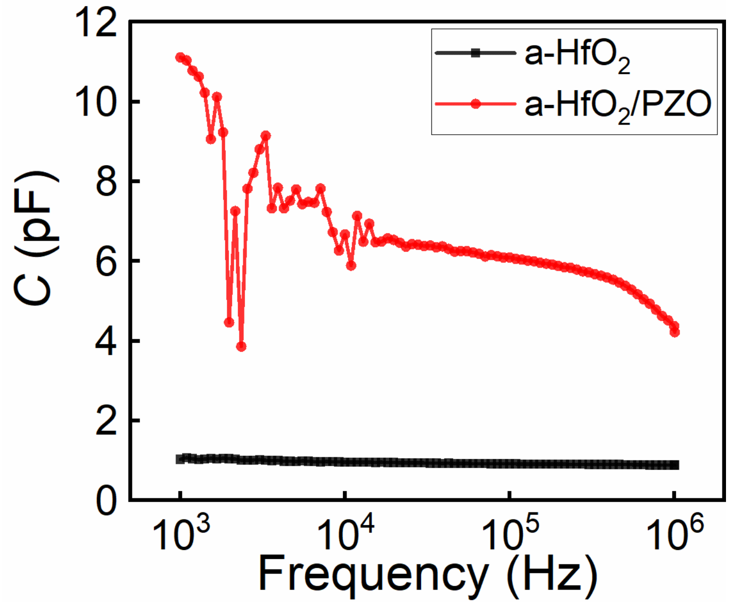

Supplementary Fig. 2 Comparison of the capacitance as a function of the frequency in Pt/a-HfO2/LSMO and Pt/a-HfO2/PZO/LSMO capacitors. The capacitance of another a-HfO2/PZO sample is indeed higher than that of the corresponding a-HfO2 sample through the whole frequency range when the frequency is swept from 1 KHz to 1 MHz.

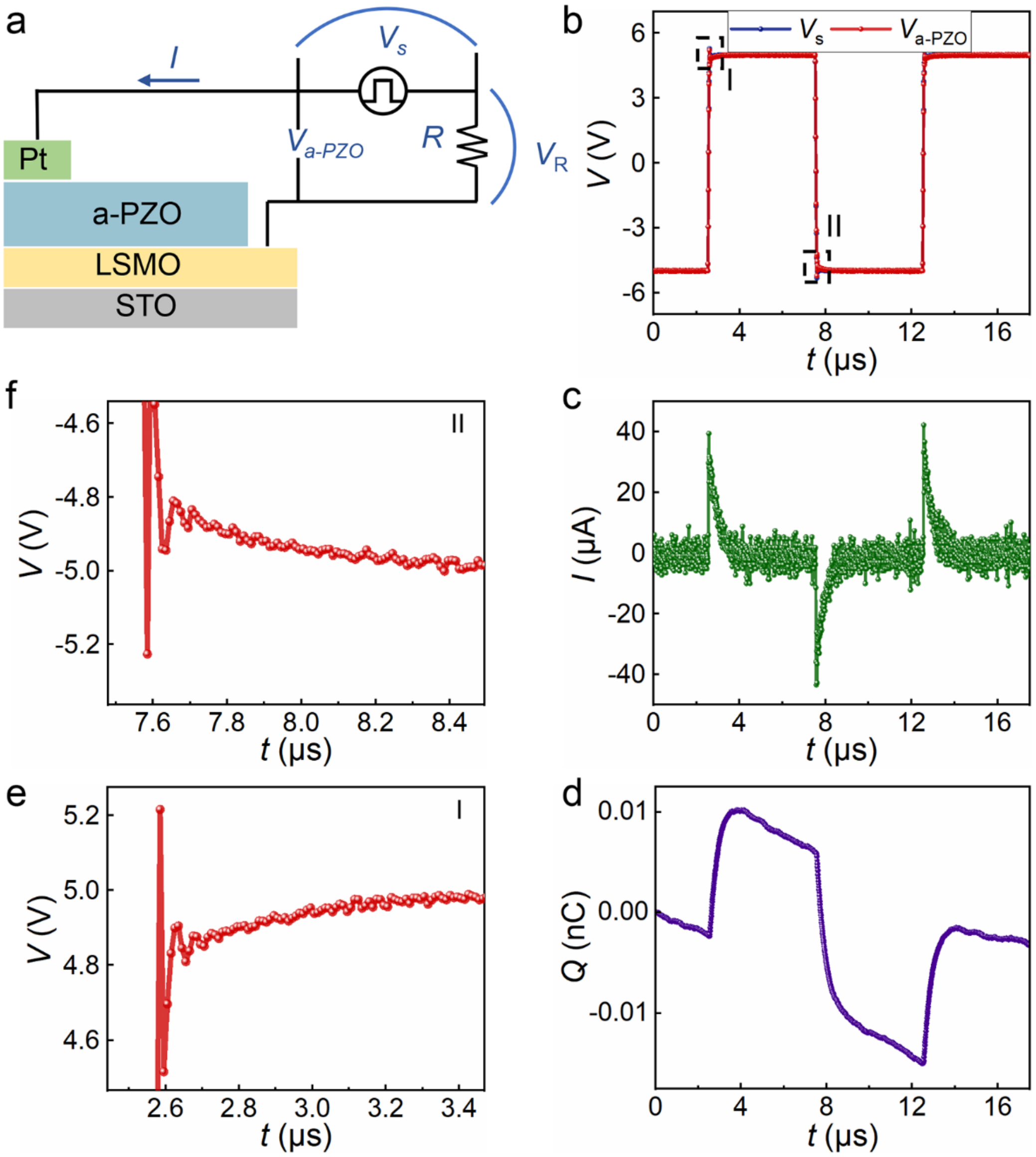

Supplementary Fig. 3 Transient response of the a-PZO capacitor. a Schematic diagram of the experimental set-up. Transients corresponding to the **b** source voltage V_s and the voltage $V_{a\text{-PZO}}$ across the a-PZO device, **c** current through the circuit, **d** charge Q on the application of an a.c. voltage pulse V_s: –5 → +5 → –5 V and R=3 KΩ. Negative capacitance transients have never been observed during the whole measurement process. The regions I and II in Fig. S3b are magnified in **e** and **f** respectively. The source voltage pulse V_s is shown as the blue line and $V_{a\text{-PZO}}$ transient as the red one.

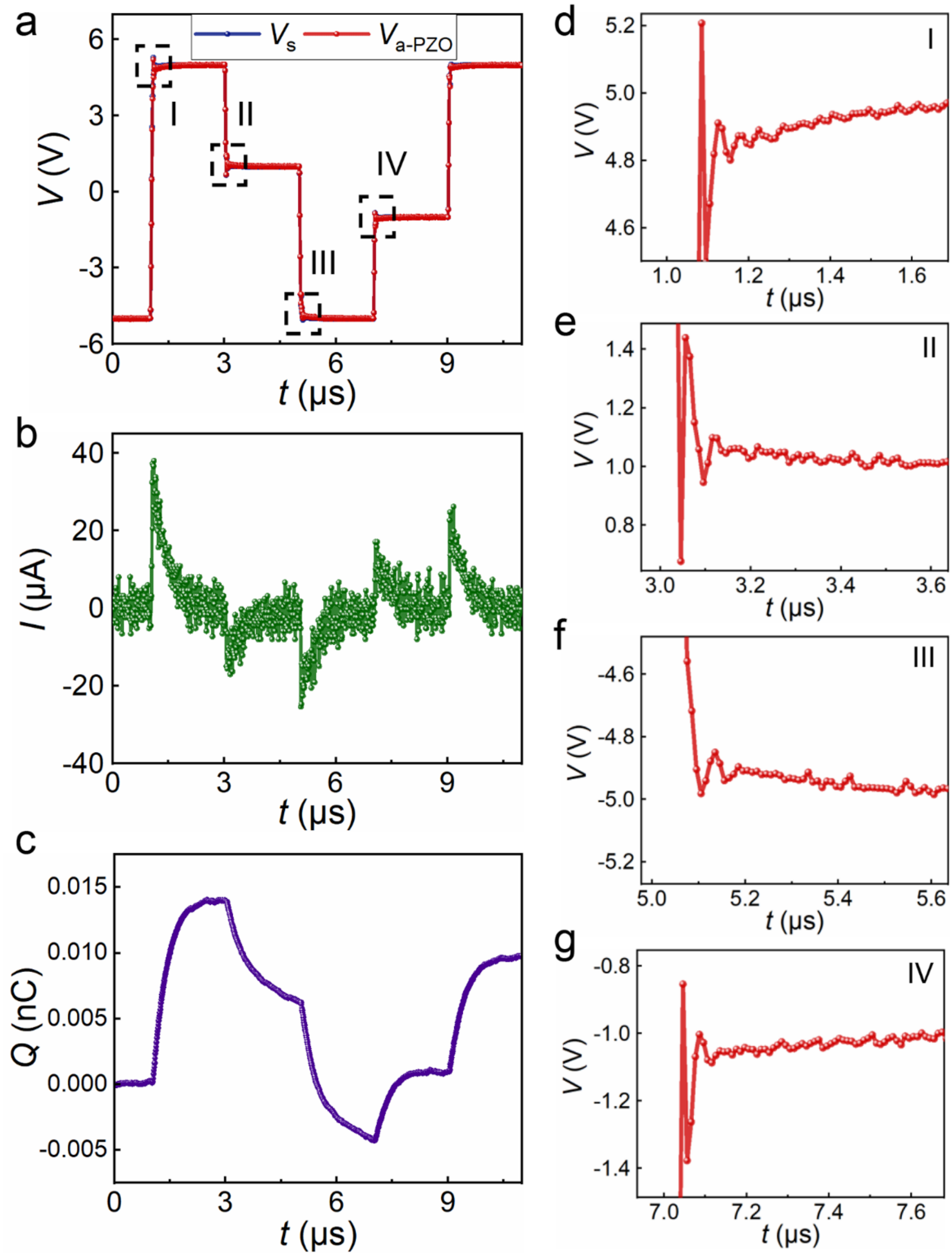

Supplementary Fig. 4 Transient response of the a-PZO capacitor under different pulse operation processes. Transients corresponding to the **a** source voltage V_s and the voltage $V_{a\text{-}PZO}$ across the a-PZO device, **b** current through the circuit, **c** charge Q on the application of an a.c. voltage pulse V_s: –5 → +5 → +1 → –5 → –1 → +5 V and R = 3 kΩ. Negative capacitance transients have never been observed during the whole measurement process.

The regions I, II, III, and IV in Fig. S4a are magnified in **d**, **e**, **f** and **g** respectively. The source voltage pulse V_s is shown as the blue line and $V_{a\text{-}PZO}$ transient as the red one.

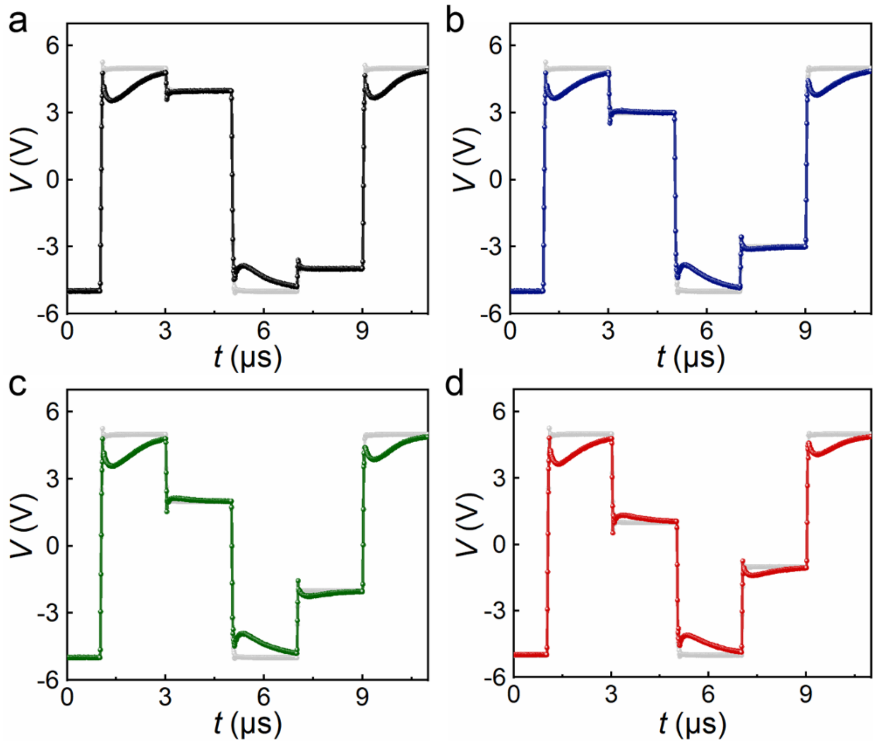

Supplementary Fig. 5 Voltage transients under different $V_{FE\text{–}AFE}$ for Pt/PZO/LSMO capacitor. Voltage transients under the voltages $V_{FE\text{–}AFE}$ of **a** 4 V; **b** 3 V; **c** 2 V; **d** 1 V with the same $V_{AFE\text{–}FE}$ of 5 V. The light grey lines represent the applied source voltage, and the colored lines represent corresponding voltage transients.

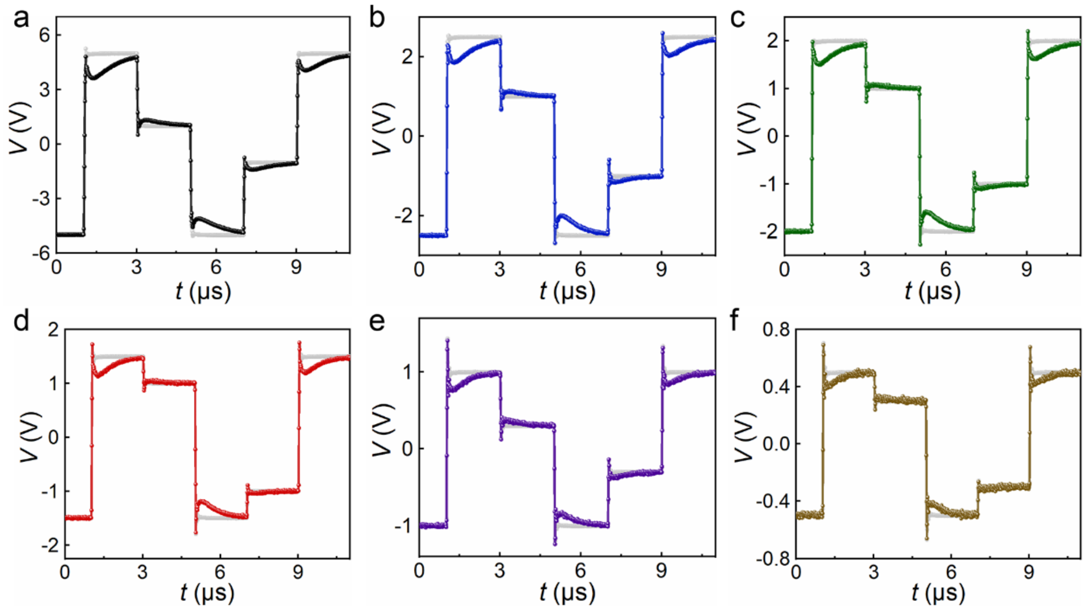

Supplementary Fig. 6 Voltage transients under different voltages $V_{AFE–FE}$ for Pt/PZO/LSMO capacitor. Voltage transients under the voltages $V_{AFE–FE}$ of **a** 5 V; **b** 2.5 V; **c** 2 V; **d** 1.5 V with the same $V_{FE–AFE}$ of 1 V. Voltage transients under the voltages $V_{AFE–FE}$ of **e** 1 V; **f** 0.5 V with the same $V_{FE–AFE}$ of 0.3 V. The light grey lines represent the applied source voltage, and the colored lines represent corresponding voltage transient.

Supplementary Note1:

In our samples, the dipoles do not arrange in a normal way like that in the bulk ceramics with antiparallel and commensurate arrangements. Two incommensurate modulations are observed in the antiferroelectric films and can interchange freely, as shown in Fig. S7. For the angle modulation mode, and the dipoles have nearly equal magnitudes, but the adjacent sublattices align with an acute angle, as displayed in Fig. S7a. For the magnitude modulation mode, the dipoles align along near the [110] and [$\bar{1}\bar{1}$0] directions, but with different magnitudes along the opposite directions, as displayed in Fig. S7b. These two unusual arrangements modes just have been observed in chemically modified PZO-based ceramics due to their smaller energy difference higher than ground state.[17,27] Here the interface limit and the strain effect make these two dipole arrangement modes stable and occur in the pure PZO film.

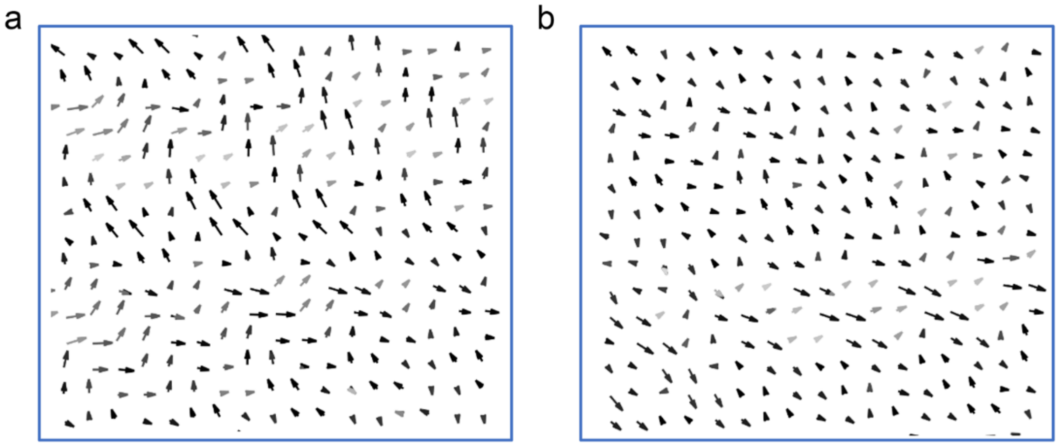

Supplementary Fig. 7 polarization vector maps showing two modulation modes. a Angle modulation mode. **b** Magnitude modulation mode.

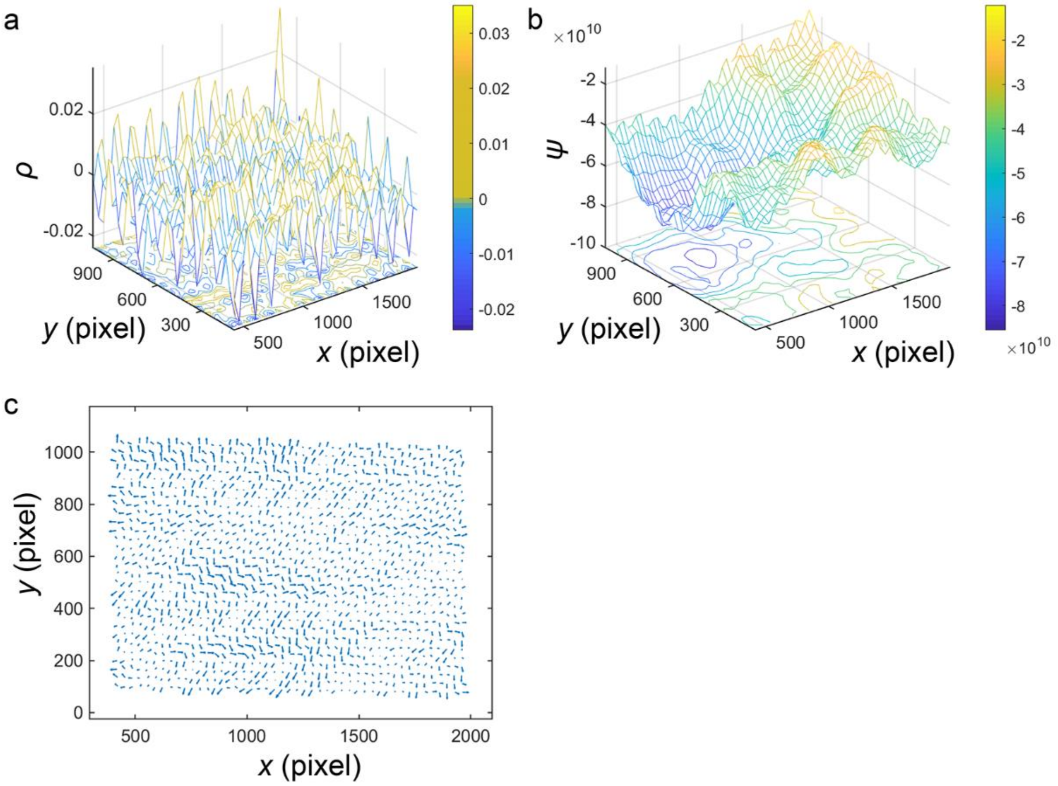

Supplementary Fig. 8 **a** Distribution of the surface density of the polarization charge ρ. **b** Distribution of the electric potential ψ. **c** Distribution of the electric displacement **D**.

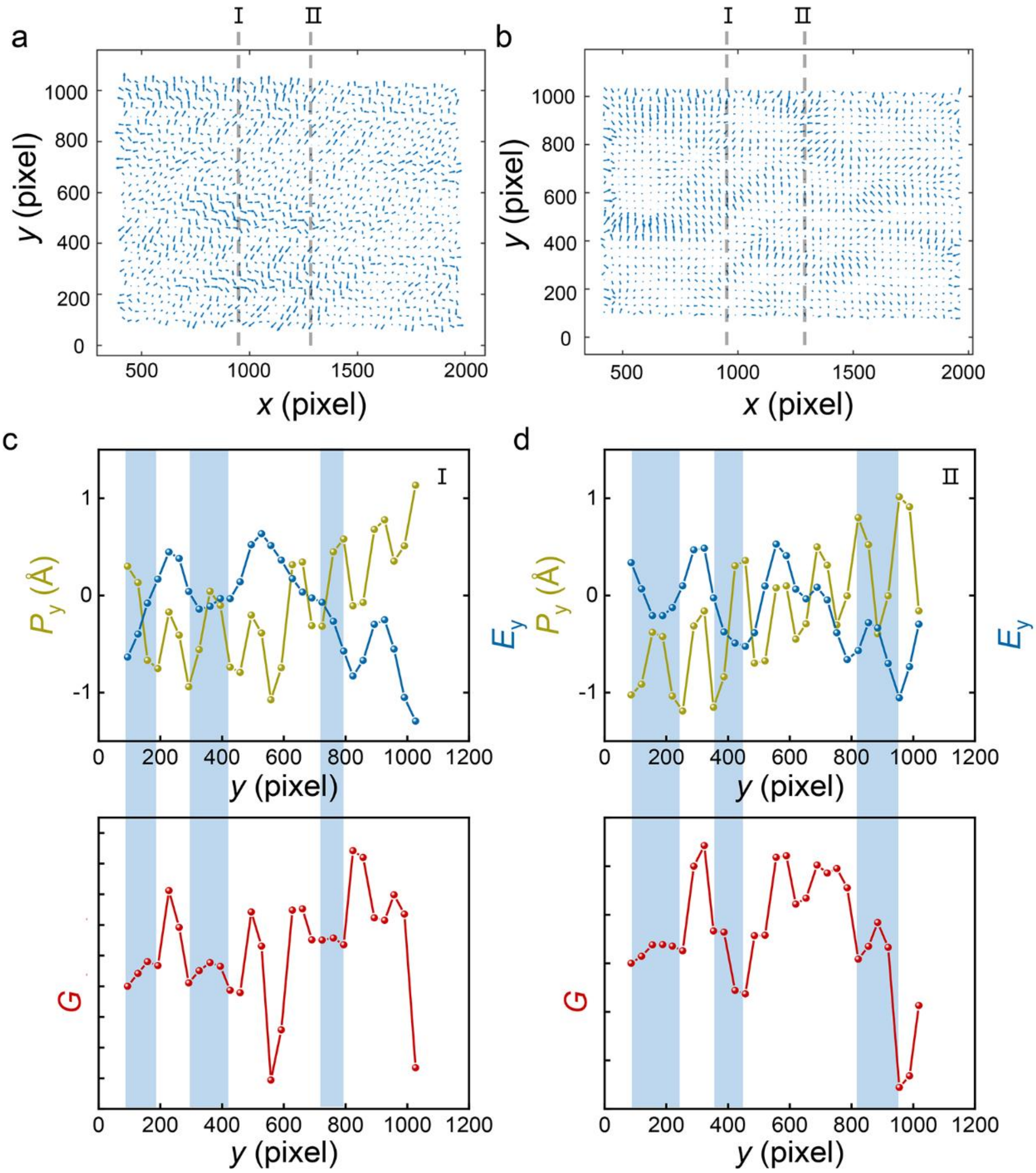

Supplementary Fig. 9 Displaying the local regions of the NC effect. a Distribution of polarization vector **P**. **b** Distribution of electric field **E** in the same region. Variation in the *y* component of the polar displacement P_y (yellow line) and the electric field E_y (blue line), and the local free energy *G* (red line) along the perpendicular lines I (**c**), II (**d**) in **a** and **b**. The light blue shadows indicate that these regions have the negative differential capacitance where $\partial^2 \mathrm{G}/\partial^2 \mathrm{D} < 0$ and $dP/dE < 0$.
